# High-Throughput Screening of Sulfur-Resistant Catalysts for Steam Methane Reforming Using Machine Learning and Microkinetic Modeling

**DOI:** 10.1021/acsomega.4c00119

**Published:** 2024-02-28

**Authors:** Siqi Wang, Satya Saravan Kumar Kasarapu, Peter T. Clough

**Affiliations:** Energy and Sustainability Theme, Cranfield University, Cranfield, Bedfordshire MK43 0AL, U.K.

## Abstract

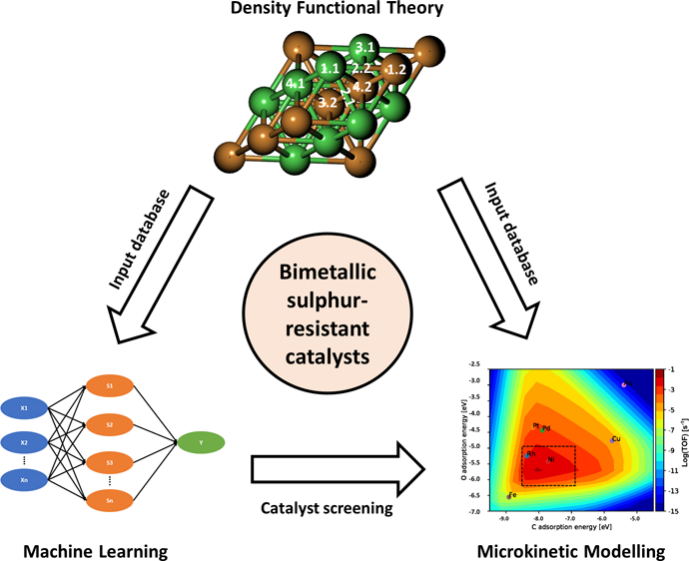

The catalytic activity of bimetallic catalysts for the steam methane reforming (SMR) reaction was extensively studied previously. However, the performance of these materials in the presence of sulfur-containing species is yet to be investigated. In this study, we propose a novel process aided by machine learning (ML) and microkinetic modeling for the rapid screening of sulfur-resistant bimetallic catalysts. First, various ML models were developed to predict atomic adsorption energies (C, H, O, and S) on bimetallic surfaces. Easily accessible physical and chemical properties of the metals and adsorbates were used as input features. The Ensemble learning, artificial neural network, and support vector regression models achieved the best performance with *R*^2^ values of 0.74, 0.71, and 0.70, respectively. A microkinetic model was then built based on the elementary steps of the SMR reaction. Finally, the microkinetic model, together with the atomic adsorption energies predicted by the Ensemble model, were used to screen over 500 bimetallic materials. Four Ge-based alloys (Ge_3_Cu_1_, Ge_3_Ni_1_, Ge_3_Co_1_, and Ge_3_Fe_1_) and the Ni_3_Cu_1_ alloy were identified as promising and cost-effective sulfur-resistant catalysts.

## 1 Introduction

Steam methane reforming (SMR) is the most widely used process for syngas production. In this process, steam and methane react in the presence of a catalyst to produce a mixture of carbon monoxide, carbon dioxide, and hydrogen. Although currently, most commercial SMR catalysts are supported nickel-based materials, extensive research on bi/polymetallic catalysts has been carried out with the aim of enhancing the overall catalytic activity and material stability.^1,2^ The stability of the catalysts is usually evaluated based on their long-term activity and resistance to metal oxidation, sintering, or undesired impurities in the reaction system. Based on a recent literature review on bi/polymetallic SMR catalysts,^3^ existing literature is mainly focused on the carbon resistance or metal oxidation resistance of the materials. Various promoters, including noble metals^4−7^ and non-noble metals,^8−10^ were found to be carbon-resistant. Noble metals were also used as promoters to improve resistance to metal oxidation^11−14^ and sintering.^15−17^

In contrast, very little literature on the sulfur resistance of SMR catalysts is available. The feed stream employed in most experimental studies is a mixture of pure methane and steam, and the effect of impurities on the performance of the catalysts was often not considered. One of the most important sources of methane—natural gas—usually contains sulfur in the form of thiophenes, mercaptans, and sulfides.^18^ Sulfur components are known to be poisonous to metallic catalysts, reducing the life of commercial catalysts to only months or weeks when ppm levels of sulfur impurities are present in the feed gas.^19^ Catalyst poisoning by sulfur usually takes place through the following processes:^20^

1. Active site blockage: an adsorbed sulfur atom may physically block a three- or four-fold adsorption site.
2. Adsorption energy modification: the chemical bond between the sulfur atom and its neighboring atom(s) can modify its ability to adsorb the reactant molecules and dissociate the product molecules.
3. Restructure of the catalyst surface: strongly adsorbed sulfur atoms can modify the surface structure and properties of the catalyst.

Adding a second metal, either as a promoter or as the second element to form an alloy with the base metal, is a commonly used method to improve the overall sulfur resistance of the catalyst. A novel Ni–Fe-based catalyst with a core–shell structure was tested by Tsodikov et al.,^21^ and its catalytic activity remained unaffected as the concentration of H_2_S in the system increased from 5 to 30 ppm. Wang et al.^22^ reported that the bimetallic Ni–Re/Al_2_O_3_ catalyst achieved a high methylcyclohexane conversion rate of 93% during the steam reforming process in the presence of 20 ppm sulfur, whereas the monometallic Ni/Al_2_O_3_ catalyst quickly deactivated. Gaillard et al.^23^ tested the stability of Ni–Mo/Al_2_O_3_ under long-term dry methane reforming conditions in the presence of 50 ppm of H_2_S and concluded that the bimetallic catalyst showed a better performance compared with the Ni or Mo counterparts. Similarly, Saha et al.^24^ reported that the addition of Co enhanced the stability of Ni/Al_2_O_3_ for the dry reforming of biogas containing 100 ppm of H_2_S. Capa et al.^25^ tested a Pd-doped bimetallic Ni–Co catalyst under sorption-enhanced steam reforming conditions and found that the catalyst was able to remain active for five cycles with an H_2_S concentration of 350 ppm.

Apart from the experimental testing of the materials of interest, microkinetic modeling (MKM) has gained increasing attention as a rapid and reliable way to evaluate heterogeneous catalysis processes.^26^ By providing the reaction mechanism and energetics (which can be obtained by experimental testing or first-principle-based calculations), the catalytic activity of any given material can be predicted as a function of simple descriptors, such as atomic adsorption energies.^27^ MKM has been successfully applied to various reforming processes for the evaluation of catalyst performance based on their composition,^28,29^ structure,^30^ and size.^31^

High-throughput screening aided by machine learning (ML) has also been applied in the rapid scanning of large material databases to find materials that may perform well based on their physical and chemical characteristics.^32,33^ Liu et al.^34^ developed an ML model to predict the adsorption energies of C and O atoms on bimetallic surfaces. With the help of a microkinetic model of the SMR reaction, they used the predicted adsorption energies to scan through over 5000 materials and identified 48 promising candidates with high SMR activity. A similar approach was adopted by Saxena et al.^35^ where the C and O adsorption energies on Cu-based bimetallic surfaces were predicted by ML and used in an MKM for ethanol decomposition. Liu et al.^36^ used ML-predicted C and O adsorption energies and the MKM for methanol synthesis, methanation, and SMR to scan over 1300 alloys in search of highly active catalysts. All of these studies focused on only the activity of the catalysts and the energetics of carbon- and oxygen-containing species. However, as mentioned previously, the stability of the materials is also an important aspect to consider, in particular their resistance to sulfur. It is therefore of interest to employ this integrated “ML + MKM” method for the rapid screening of sulfur-resistant catalysts.

In this study, various ML algorithms were used to predict the adsorption energies of C, O, H, and S atoms on monometallic and bimetallic surfaces, using readily available physical and chemical properties as input features and density functional theory-calculated adsorption energies as the target values. A microkinetic model of the SMR reaction was developed, together with the ML-predicted energies. These were used for the scanning of over 500 bimetallic alloys. To the best of the authors’ knowledge, this is the first-ever attempt at the systematic high throughput screening of sulfur-resistant SMR catalysts.

## 2 Methods

### 2.1 Density Functional Theory

All DFT-based calculations in this work were carried out using the Quantum Espresso software.^37^ The core electrons were described by using the Kresse-Joubert Projector Augmented Wave (PAW) method. The exchange correlations were described using the generalized gradient approximation with the Perdew–Burke–Ernzerhof functional.^38^ The cutoff energies for the wave function and charge were set to 25 and 250 Ry, respectively. The convergence criteria for force and energy were set to be 0.025 eV/Å and 10^–5^ eV, respectively. All catalysts were simulated by a four-layer *p*(2 × 2) slab model, with the top two layers and the adsorbate relaxed and the bottom two layers fixed. The k-point grid was set to be 3 × 3 × 1 for the sampling of the Brillouin zone. A 10 Å vacuum was added to separate the two neighboring layers in the *z*-direction. The adsorption energy (*E*_ads_) is calculated as below:
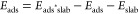
1where *E*_ads*slab_ is the total energy of the slab with the adsorbate, *E*_ads_ is the total energy of the gas phase adsorbate, and *E*_slab_ is the total energy of the clean slab.

Adsorption on the following close-packed surfaces was considered: [111] for face-centered cubic (FCC) systems (e.g., Ni, Cu, Rh, all bimetallic systems, etc.), [110] for body-centered cubic systems (e.g., Fe, Nb, Mo, etc.), and [0001] for hexagonal close packing systems (e.g., Co, Zn, Ru, etc.). All bimetallic alloys were considered to be FCC systems. For the monometallic FCC surfaces, four adsorption sites were considered, namely, top, bridge, FCC, and hexagonal close-packed sites. For the bimetallic surfaces, eight high-symmetry adsorption sites were considered. The precise locations of the adsorption sites are illustrated in the Supporting Information.

In the case where multiple adsorption sites are available, the site with the lowest adsorption energy was chosen, as it indicates the most stable geometry.

All gas phase species were modeled by placing the molecule in a cube with the lattice parameters of *a* = 20 Å, *b* = 20.5 Å, *c* = 21 Å. The k-point grid was set to be 1 × 1 × 1 for all gas phase calculations.

### 2.2 Machine Learning

#### 2.2.1 Database Construction

The database used for the ML model training consists of DFT-calculated adsorption energies of C, H, O, and S on 23 monometallic and 12 bimetallic surfaces (a total of 140 data points). Each pure metal is represented by a set of 12 features, including fundamental properties (e.g., group, atomic number, covalent radius, etc.) and surface-related properties (e.g., surface free energy, work function, etc.). Each alloy (M1_*x*_M2_*y*_) is represented by the features of its components (12 features of M1 plus 12 features of M2) and the ratio of *x*:*y* to account for the concentration of each component within the binary system. For monometallic inputs, the ratio was considered as 1. The adsorbates (C, H, O, and S) are represented by a set of nine properties, including group, atomic number, first ionization potential, etc. The complete input database, including numerical values of the features and the adsorption energies, can be found in the Supporting Information.

#### 2.2.2 ML Algorithms

Different ML algorithms were used for the prediction of C, H, O, and S adsorption energies including linear regression (LR), ridge regression (RR), K-nearest neighbors (KNN) regression, random forest regression (RFR), extra trees regression (ETR), gradient boosting regression (GBR), support vector regression (SVR), light gradient boosting machine (light GBM), artificial neural network (ANN), and Ensemble learning.

The ANN model was implemented using Keras with a TensorFlow backend.^39^ All other algorithms were implemented using the open-source ML library Scikit-Learn.^40^ The data set was randomly split into training (80%) and testing (20%) subsets. The accuracy of the models was evaluated based on the mean-squared error (MSE), mean absolute error (MAE), and coefficient of determination (*R*^2^). The evaluation metrics were calculated using the following equations:
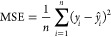
2
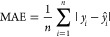
3
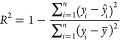
4where *n* is the number of samples, *y*_*i*_ the DFT-calculated value of sample *i*,  the predicted value of sample *i*, and the *y̅* the average of the DFT-calculated values.

Tuning of the hyperparameters was conducted using the five-fold cross-validation (CV) method via GridSearchCV. The range of hyperparameters tested for each model during the tuning process is summarized in Table 1. Feature importance study was carried out using recursive feature elimination. The features were ranked based on their importance, and the ones that did not contribute significantly to the model performance were eliminated from the model.

**Table 1:** Range of Hyperparameters Tested for ML Models

ML algorithm	hyperparameters
RR	alpha = [0.5, 0.75, 1, 1.25, 1.5]
KNN	N neighbors = [1, 3, 5, 7, 9]; weights = [uniform, distance]
RFR	max depth = [25, 50, 75, 100]; N estimators = [500, 1000, 1500, 2000]
ETR	max depth = [25, 50, 75, 100]; N estimators = [500, 1000, 1500, 2000]
GBR	max depth = [25, 50, 75, 100]; N estimators = [500, 1000, 1500, 2000]; learning rate = [0.01, 0.03, 0.05, 0.07]
SVR	kernel = [“linear”, “rbf”]; C = [0.1, 1, 10]
light GBM	max depth = [25, 50, 75, 100]; N estimators = [500, 1000, 1500, 2000]; learning rate = [0.01, 0.03, 0.05, 0.07]
ANN	number of layers = [2, 3, 4, 5]; dropout rate = [0.1, 0.2, 0.3, 0.4, 0.5]; learning rate = [0.01, 0.001, 0.005]; epochs = [100, 200, 300, 400, 500]

### 2.3 Microkinetic Modeling

The MKM was implemented with the descriptor-based analysis tool CatMAP,^41^ which provides a flexible and automated framework for constructing descriptor-based microkinetic analyses, and the production rate of a defined system is calculated as the turnover frequency (TOF). The model of the SMR reaction was developed based on seven pure transition metals that have been widely used as catalysts or catalyst promoters for the SMR reaction: Rh, Ni, Cu, Fe, Pd, Pt, and Au. DFT-calculated energies were used as the model input together with energetics calculated and estimated using the unity bond index-quadratic exponential potential (UBI-QEP) method and the Bro̷nsted–Evans–Polanyi (BEP) relationship. These methods have been widely used in the field of surface adsorption and are capable of yielding accurate results without the need for time-consuming first-principle-based calculations.^42−44^ Detailed information on the input data is available in the Supporting Information. A total of 12 elementary steps were considered in the MKM, including CH_4_ and H_2_O dehydrogenation, CO formation through multiple routes (COH* and HCO* dehydrogenation and CO* desorption), and interaction between various intermediate species. A full list of the elementary reaction steps can be found in Supporting Information. It should be noted that although the actual reaction mechanism of SMR is more complicated with other intermediate species involved, the simplified mechanism used in this work is accurate enough to describe the overall trend of the reaction without overly high computational demand.^29^ The reaction conditions were set to be 1073 K and 1 bar, with a H_2_O:CH_4_ ratio of 3, which are the typical conditions used for the SMR process. The Shomate equation and frozen adsorption were used to model the gas-phase species and adsorbates, respectively. The Shomate parameters for the gases were obtained from the NIST Web site.^45^

To conclude the methodology used in this work, a combined “ML + DFT + MKM” approach was employed, including the following steps:

1. DFT calculations of C, O, H, and S adsorption energies were carried out for 35 metallic surfaces.
2. Part of the DFT-calculated energies were used for the construction of the microkinetic model.
3. All DFT-calculated energies were used as the input data set for the ML models.
4. The best-performing ML model was used to predict the C, O, H, and S adsorption energies on over 500 bimetallic surfaces.
5. The 500+ bimetallic materials were screened based on results from the microkinetic model.

**Figure 1:**
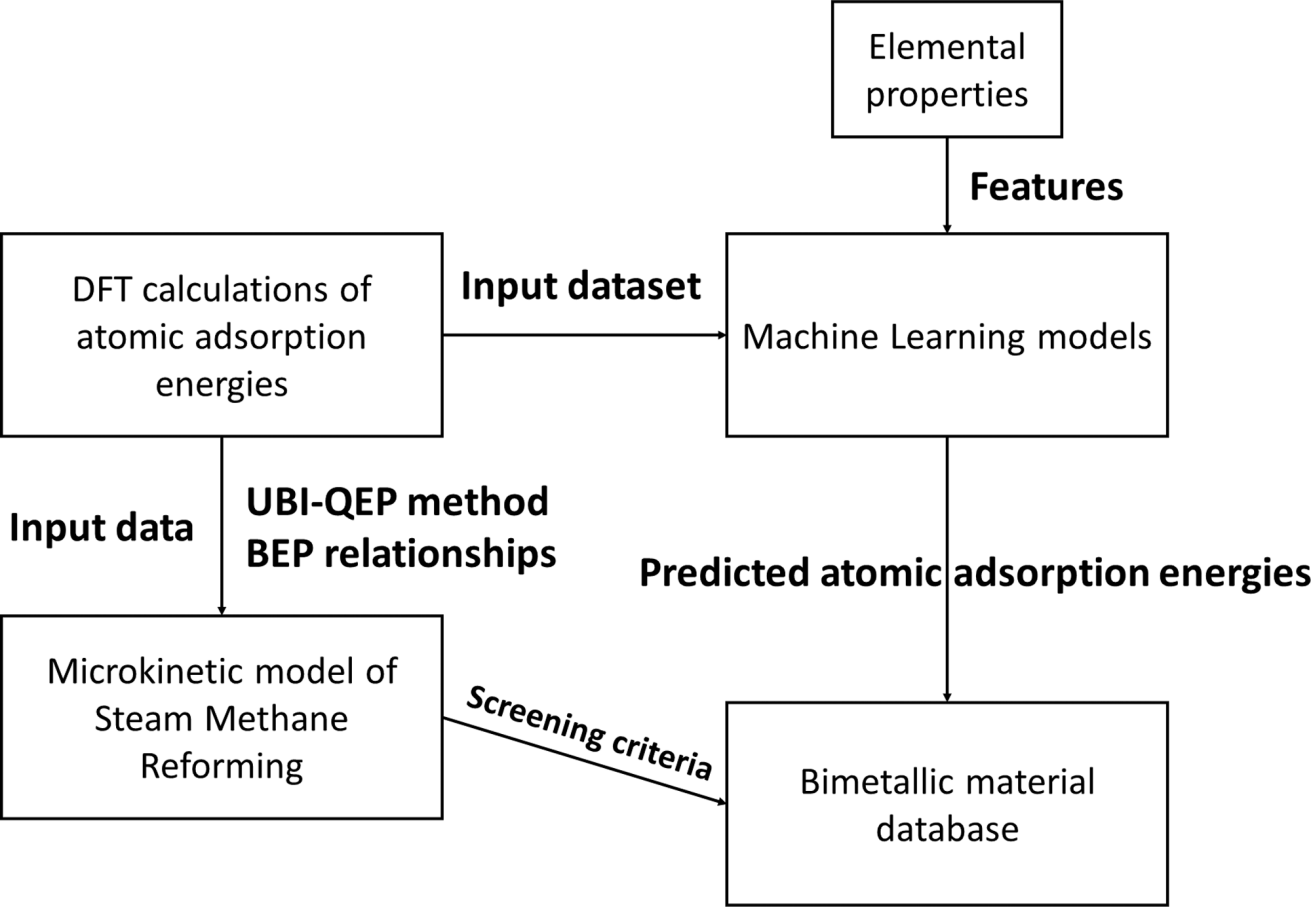
Schematic diagram of the “ML + DFT + MKM” approach.

A schematic diagram of the methodological approach employed is provided in Figure 1.

## 3 Results and Discussion

### 3.1 ML Models

#### 3.1.1 Hyperparameter Tuning

Hyperparameter tuning is an essential step in the ML model development process; it ensures that the model developed is complex enough to capture the characteristics of the input features but at the same time not too complex to cause overfitting. The types of hyperparameters tuned and the range of values before and after the tuning process are summarized in Tables 1 and 2, respectively.

**Table 2:** Optimized Hyperparameters for Each ML Algorithm

ML algorithm	hyperparameters
RR	alpha = [1.25]
KNN	N neighbors = [5]; weights = [distance]
RFR	max depth = [75]; N estimators = [2000]
ETR	max depth = [100]; N estimators = [1500]
GBR	max depth = [100]; N estimators = [1000]; learning rate = [0.07]
SVR	kernel = [“rbf”]; C = [10]
light GBM	max depth = [25]; N estimators = [500]; learning rate = [0.01]
ANN	number of layers = [3]; dropout rate = [0.2]; learning rate = [0.001]; epochs = [500]

#### 3.1.2 Feature Selection and Engineering

Feature selection is an important step in the development of the ML model development process. The features selected should be unique in representing the geometric and electronic structures of a specific adsorption site. Commonly used features can be divided into three main categories:

1. Elemental properties include atomic number, mass and radius; ionic potential; electronegativity, etc. These properties only depend on the host–metal atom and can be easily obtained from literature or the periodic table.
2. Electronic properties include d-band features such as center, filling, width, skewness, kurtosis, etc.^46−48^ These features are more complex than basic elemental properties and usually require single-point DFT calculations to obtain.
3. Geometric properties include local electronegativity and effective coordination number.^49,50^ These features are usually used when the adsorption energies on different adsorption sites are compared. This is not the case for this study, as the most stable adsorption sites have been preselected during the DFT calculation process.

In this work, only the elemental properties were considered as input features, as they are readily available and have been shown to produce sufficiently accurate results.^51−54^ As mentioned in Section 2.2.1, 12 features for each metal, 9 features for each adsorbate, and the ratio between the two metals were selected as the initial input features. This means that 21 features were used to represent one atomic adsorption energy on a monometallic surface and 34 features on a bimetallic surface. However, as we were dealing with a relatively small database (less than 200 data points in the input database), a large number of input features may lead to overfitting. It is therefore necessary to identify and eliminate features with diminished importance that may contribute to inaccuracies during the prediction process.

**Figure 2:**
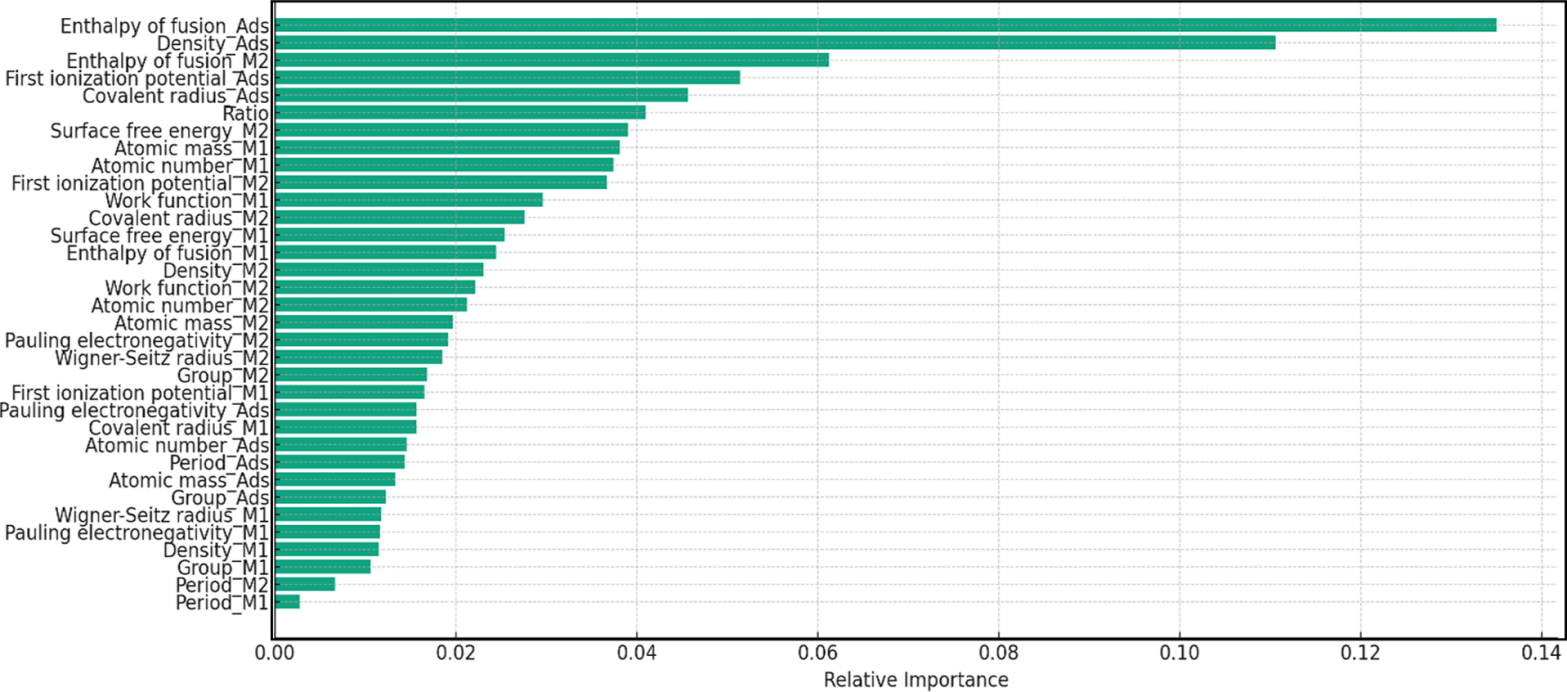
Relative feature importance for the Ensemble model.

Feature importance was evaluated using the Ensemble model with tuned hyperparameters, and the relative importance of each feature is presented in Figure 2. The Gini importance parameter was used to evaluate the importance of each feature (eq 5):
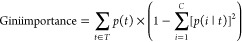
5where *t* is the set of all nodes that use the given feature, *p*(*t*) is the proportion of samples reaching the node *t*, and *p*(*i*|*t*) is the proportion of class samples *i* at node *t*.

The result shows that the properties of the adsorbate contribute most significantly to the prediction, with four out of the top five features being the enthalpy of fusion, density, first ionization potential, and covalent radius of the adsorbate. Properties of the metals, including enthalpy of fusion, surface free energy, atomic mass, atomic number, and first ionization potential, were also found to have relatively high importance. This is consistent with the trend observed by Nayak et al.,^55^ where the most important features are first ionization potential and enthalpy of fusion for the absorbate, and group, surface free energy, and enthalpy of fusion for the surface. In order to enhance the overall accuracy of the ML prediction, only the top 12 features were included for the models. This ensures that the predictions are made based on the most informative and pertinent features and therefore deliver the most precise predictions of the adsorption energies.

**Figure 3:**
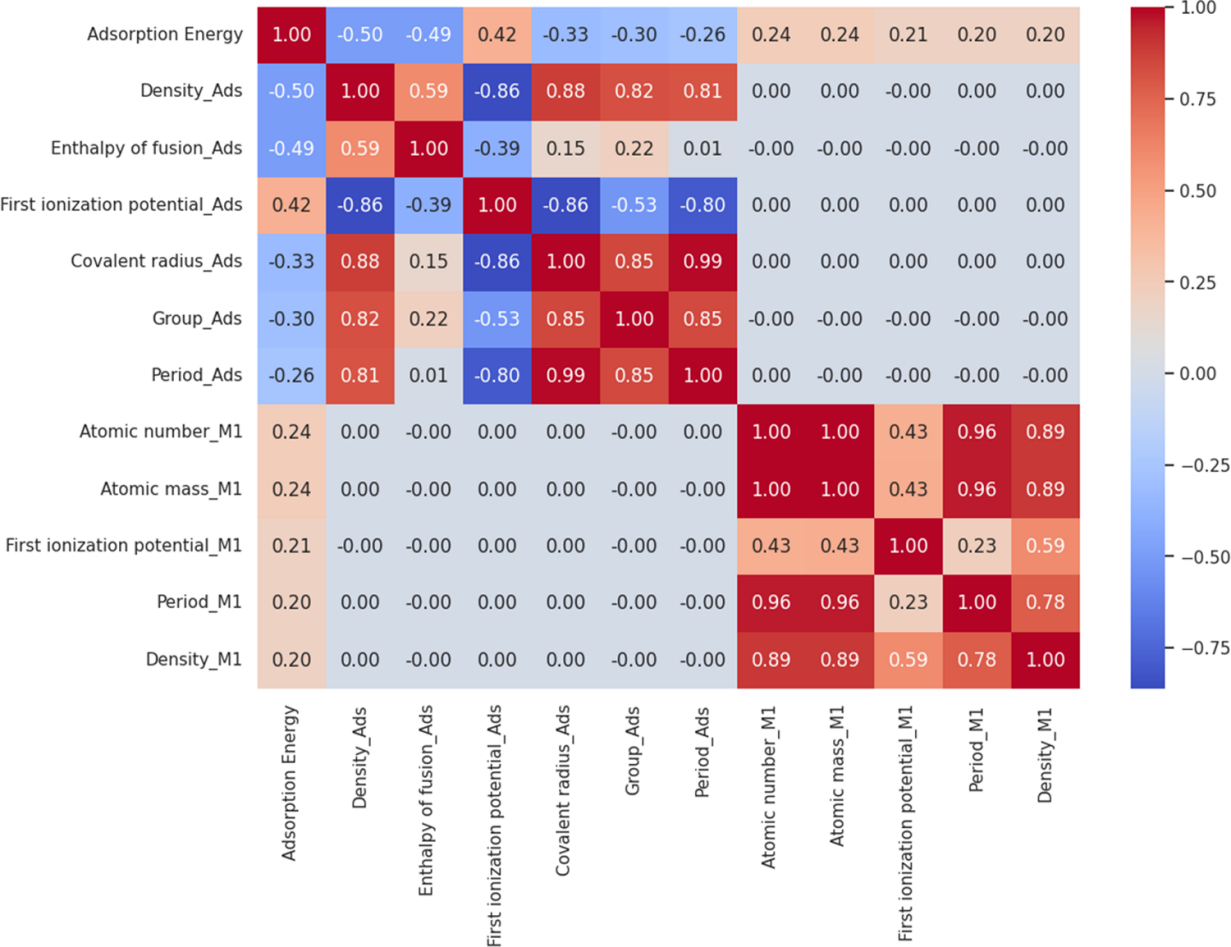
Correlation heatmap for the adsorption energy with the top 11 features.

The linear relationship between each of the 11 features (top 12 most important features, excluding “ratio”) and the target value, adsorption energy, was quantified by the Pearson correlation coefficient. The result is presented in the form of a heatmap in Figure 3. The first ionization potential of the adsorbate exhibits the highest positive correlation with a correlation coefficient of 0.42, which again confirms the significance of the adsorbate’s electronic properties. This is consistent with the previous observations that higher charge transfer leads to lower adsorption energy.^56^ A higher ionization potential represents a lower probability for charge transfer and therefore a higher adsorption energy. The density of the adsorbate exhibits the most substantial negative correlation with a correlation coefficient of −0.5. This is in line with the general trend that heavier atoms tend to have lower adsorption energies. For instance, the adsorption energies of carbon on transition metal surfaces are usually in the range of −5.0 to −10.0 eV, whereas the adsorption energies of hydrogen are in general between −2.0 and −5.0 eV.

#### 3.1.3 Model Performance Evaluation

**Figure 4:**
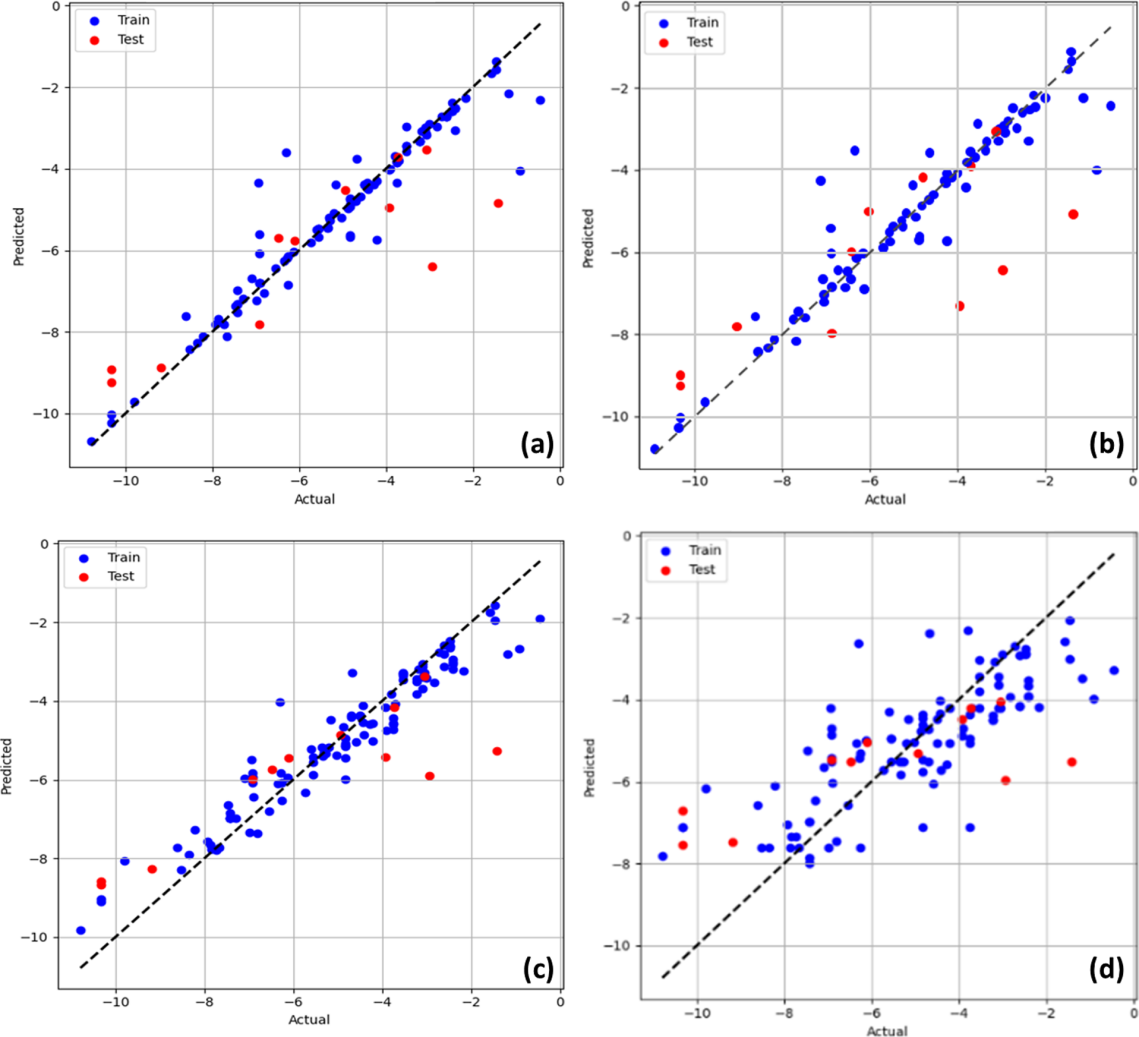
Regression plots for the three best-performing models: (a) ER, (b) ANN, (c) SVR, and (d) the low-performing KNN model.

Following the model optimization process, all ML models were reprogrammed by using the top 12 features and the tuned hyperparameters. The evaluation metrics obtained for each model, including train and test MSE, MAE, and *R*^2^, are summarized in Table 3. The regression plots of the three best-performing models (Ensemble, ANN, and SVR) and the low-performing KNN model are presented in Figure 4. The ensemble model exhibited the best overall performance with the highest test *R*^2^ of 0.74, a test MSE of 1.45, and a test MAE of 0.76. This was followed by the ANN and SVR models with test *R*^2^ values of 0.71 and 0.70, respectively. On the other hand, the KNN model produced the least satisfying results, with the lowest test *R*^2^ of 0.48 and the highest MSE of 3.42. It can be observed from the regression plot (Figure 4d) that the KNN model presented a random pattern of predictions with dispersed training and testing results. This is possibly due to the localized focus of the KNN model, which does not suit the complexity of the data set.

**Table 3:** Performance Evaluation Metrics of the ML Models

model	train			test		
	MSE	MAE	*R*^2^	MSE	MAE	*R*^2^
Ensemble	0.37	0.38	0.92	1.45	0.76	0.74
ANN	0.42	0.43	0.91	1.93	0.79	0.71
SVR	0.44	0.40	0.91	1.19	0.80	0.70
RFR	0.32	0.39	0.93	1.96	0.81	0.69
light GBM	0.92	0.68	0.78	2.68	1.23	0.59
ETR	0.93	0.72	0.76	2.72	1.27	0.58
GBR	0.98	0.72	0.75	2.78	1.31	0.57
RR	1.79	0.92	0.63	2.90	1.34	0.56
LR	1.44	0.79	0.70	1.74	1.41	0.52
KNN	1.84	0.93	0.61	3.42	1.56	0.48

The ensemble, ANN, and SVR models demonstrated their ability to capture the underlying patterns of the data set effectively. The models are able to produce accurate predictions for adsorption energies in the range of −4.0 to −8.0 eV. However, the models faced challenges when encountering extreme values (i.e., adsorption energies higher than −4.0 eV and lower than −8.0 eV), leading to an increase in the MSE values. This is not uncommon, as the performance of ML models is sometimes limited when the values deviate significantly from the data set’s central tendencies. As the adsorption energies of the C, H, O, and S atoms on most transition metal surfaces usually reside in the range −4.0 to −8.0 eV, it is physically unlikely to further increase the amount of input data outside this range.

Another possible reason for the models’ inability to achieve a significantly higher *R*^2^ value (*R*^2^ > 0.9) is the high dimensionality of the relatively small data set and potential feature redundancy, which usually leads to overfitting.^57^ In this work, more than 20 features were initially used, and the top 12 features were retained after the feature importance study. Although no direct linear relationship exists between each of the 12 features, some may exhibit a correlation. For example, elements with stronger intermolecular forces (higher enthalpy of fusion) may tend to have higher densities due to tighter packing in the solid state.^58^ Redundant features increase the complexity of the model without adding significant new information. Further optimization of the input features selection process can be carried out using techniques such as genetic algorithms^59^ to determine both the optimal features and optimal number of features.

It should also be noted that the accuracy of ML models relies heavily on the size of the input database. The performance of the models developed in this work can be further optimized by conducting additional DFT calculations to increase the number of input data points. This is further addressed in Section 3.3, where the limitations of the models are analyzed and suggestions for future improvement are given.

#### 3.1.4 Predicted Adsorption Energies

The best-performing ML model was then applied to a list of bimetallic alloys, of which the adsorption energies were not all readily available. A total of 24 metal elements were considered and permuted with one another, which generated a set of over 500 (24 × 23 = 552) bimetallic alloys. As mentioned in Section 2.2, one of the input features used for the ML model is the ratio of the two individual components within the binary system. By changing the numerical value of the “ratio” feature, the ML model is able to deal with a given binary alloy with any M1 or M2 concentration. In this work, we focused on bimetallic materials with a M1:M2 ratio of 3 (i.e., 75 mol % of M1 and 25 mol % of M2).

**Figure 5:**
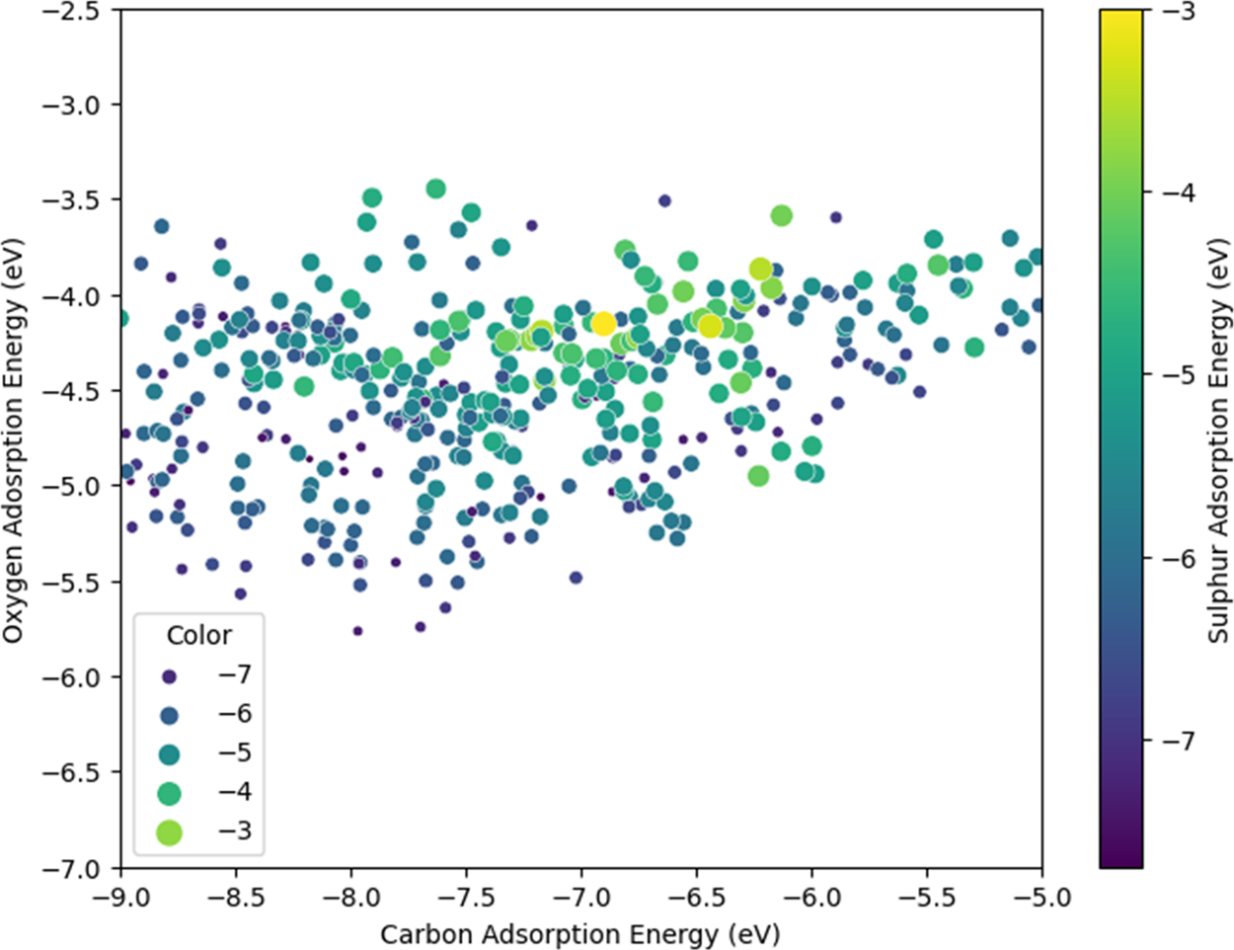
Scatter plot of C, O, and S adsorption energies of 500+ bimetallic catalysts. (Smaller circles with darker blue color represent lower S adsorption energies, and bigger circles with light yellow color represent higher S adsorption energies.)

The predicted C, O, and S adsorption energies are visualized in a scatter plot (Figure 5). The complete list of predicted energies is available in the Supporting Information. The *X* and *Y* axes of the plot represent the C and O adsorption energies, respectively. The S adsorption energies are displayed using color coding, where a darker blue color represents a lower value (therefore more prone to S adsorption) and a lighter green represents a higher value (less prone to S adsorption). Based on previous research on the interaction between sulfur and transition metal-based catalysts,^60,61^ sulfur poisoning takes place through two main routes: the reversible adsorption of sulfur-containing species (at lower concentrations) and the irreversible chemisorption of sulfur due to the formation of metal sulfides. Therefore, having a higher value of S adsorption energy is in general favorable for the catalyst, as it allows for a more difficult adsorption and easier desorption of sulfur species. A trend that can be observed from the scatter plot is that materials with higher S adsorption energies tend to have higher C and O adsorption energies, as well. However, unlike the S adsorption energy, having higher C and O adsorption energies is not necessarily beneficial for the SMR reaction to take place. A material’s catalytic activity for the SMR reaction is highly dependent on the C and O adsorption energies.^28,29,34^ Based on the Sabatier principle,^62^ the binding abilities of the catalyst should be neither too strong for the products to desorb, nor too weak for the reactants to adsorb. It is therefore crucial to identify the materials that have high sulfur resistance and, at the same time, appropriate C and O adsorption abilities for good catalytic activity.

### 3.2 MKM and Catalyst Screening

**Figure 6:**
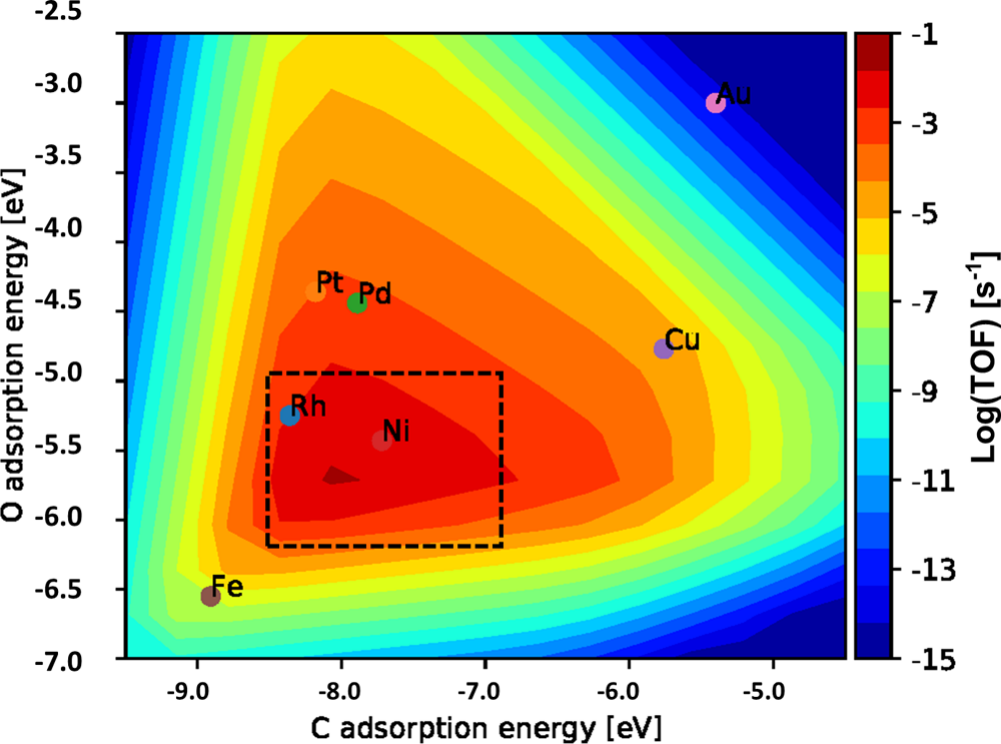
Volcano plot of the SMR model (boxed area: highest catalytic activity).

In order to identify the optimal C and O adsorption energies, an MKM of the SMR reaction was developed, and the results obtained are presented in the form of a “volcano plot” (Figure 6). The *X* and *Y* axes indicate the C and O adsorption energies, respectively. The color of the contours indicates the TOF of the production of hydrogen, which is directly linked to the catalytic activity of the catalysts. The C and O adsorption energies of the seven pure metals are in the range of [−8.9, −5.5] and [−6.2, −2.9] eV. The activity trend of the materials is in the order of Rh > Ni > Pd ≈ Pt > Fe > Au, which is consistent with the general trend observed for these metals.^63,64^ The boxed area in Figure 6 represents the region for the highest catalytic activity; the optimal C and O adsorption energies were therefore identified to be C [−8.5, −7.0] eV and O [−6.5, −5.0] eV. The database containing ML-predicted adsorption energies of the bimetallic alloys was then scanned through, and the materials that were located in the optimal range were considered to be highly active catalysts for SMR.

A total of 49 bimetallic alloys were identified as having optimal C and O adsorption energies for the SMR reaction (Table S4). These candidates were then sorted based on their S adsorption energies, and the top 10 materials with the highest S adsorption energy were considered the most promising sulfur-resistant SMR catalysts. Among the 10 catalysts, we have identified one Tc-based alloy (Tc_3_Ni_1_), three noble metal-based alloys (Rh_3_Co_1_, Rh_3_Fe_1_, and Ru_3_Cu_1_), one Ni-based alloy (Ni_3_Cu_1_), and five Ge-based alloys (Ge_3_Cu_1_, Ge_3_Ni_1_, Ge_3_Co_1_, Ge_3_Pd_1_, and Ge_3_Fe_1_). Due to the carcinogenicity and radioactivity of Pd and Tc, Ge_3_Pd_1_ and Tc_3_Ni_1_ are not considered as suitable candidates. Rh- and Ru-based materials have been proven to have excellent catalytic activity and coke resistance for SMR.^12−14^ The long-term stability of noble metal-based catalysts has also been investigated. A series of supported noble metal-based catalysts (including Ru and Rh) were tested with daily start-up and shut-down cycles under SMR conditions^12,65,66^ and the catalysts remained stable without suffering from deactivation due to sintering or metal oxidation. However, not as many studies have been carried out to investigate the sulfur resistance of these noble metal-based materials. A CeO_2_–Al_2_O_3_-supported Rh–Ni catalyst was reported to maintain a high conversion of 95% for 72 h during the reforming process of a jet fuel containing 22 ppm sulfur.^67,68^ This was attributed to the migration of S in RhS_*x*_ species to Ni in the close vicinity of Rh, due to the sulfur spillover effect. Ni–Cu-based bimetallic catalysts have been tested for various reforming processes^69−71^ and have been proven to achieve enhanced activity compared to their individual Ni and Cu counterparts. The stability of bimetallic Ni–Cu catalysts was evaluated in terms of their carbon resistance and long-term stability. Khzouz et al.^69,72^ carried out a 20-h SMR test with bimetallic Ni–Cu/Al_2_O_3_ catalysts, and no significant decrease in hydrogen yield was observed. This was attributed to the stabilizing effect from NiCu alloy formation, where the large Ni particles prone to sintering and carbon deposition were diluted by the Cu atoms. However, no experimental evaluation of their sulfur resistance is currently available. Compared to transition metal-based catalysts, the use of Ge-based materials as catalysts is less common, and their performance under SMR conditions is less well understood. Ge-based bimetallic catalysts have been tested for hydrogen production from ammonia borane^73^ and hydrogenation of acetylene^74^ and have shown enhanced activity and selectivity. Although no experimental validation is available, the sulfur-resistant ability of germanium may be attributed to its electronic structure. The electronic structure of sulfur is very similar to tetra-valent metals, including germanium, which has spare p-electrons in its outer shell next to a stable s-orbital. The interaction between the spare 4p electrons from sulfur and the metal atoms leads to the formation of metal sulfides.^75^ It is therefore possible that the p-electrons from Ge act as a “placeholder” on the bimetallic surface and block the route for potential bond formation between sulfur and the other metallic elements.

### 3.3 Limitations and Future Outlook

In this section, we aim to provide an analysis of the proposed “ML + MKM” screening method and suggestions for further improvement. First of all, it is crucial to acknowledge the limitations of using a relatively small database in the context of ML. The quantity and quality of the input data have always been one of the greatest challenges when dealing with adsorption energy prediction, particularly due to the following reasons:

1. Lack of data for a specific atom/molecule: C and O are two of the most studied atoms in the field of heterogeneous catalysis and information on their adsorption abilities is readily available from commonly used databases (e.g., Catalysis Hub,^76^ CatApp,^77^ etc.). However, data on sulfur adsorption energy are less accessible, and DFT-based calculations are therefore usually inevitable.
2. Lack of adsorption data on uncommon surfaces: Data available from the previously mentioned databases is usually focused on group VIII–XI metal elements only. DFT calculations are required to obtain information on metalloid surfaces or other uncommon transition/post-transition metal surfaces.
3. Heavy computational burden: First-principle-based DFT calculations demand very high computational capacity and can be time-consuming. For instance, the computational time needed for a single adsorption energy calculation is approximately 100 min on eight high-performance CPUs.^35^

By an increase in the size of the input database, it is possible to further improve the accuracy of the ML predictions. It is also recommended that more bimetallic alloys are added to the training base so that the models can better capture the full complexity and variability of the adsorption process on bimetallic surfaces. The MKM can also be improved by refining the reaction mechanism described in the model. For instance, the water–gas shift reaction (CO + H_2_O ↔ CO_2_ + H_2_) can be added as it takes place simultaneously with the SMR reaction under realistic experimental conditions. Reactions involving sulfur-containing species can also be added to the MKM. Taking H_2_S as an example, the interaction between the sulfur-containing species, the catalyst surface, and other reaction intermediates can be summarized by the following reaction steps:^78^
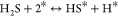

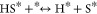

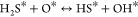

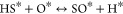

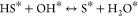

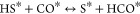

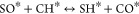


However, it should be noted that by increasing the number of reactions and intermediate species, more DFT calculations are consequently needed. It would also be of interest to utilize ML for other energy-related predictions. In this work, we used sulfur adsorption energy as a simple indicator of the material’s sulfur-resistant ability. However, other parameters such as the activation barrier of the H_2_S decomposition can also serve as accurate indicators. Similarly, the use of ML to predict these activation barriers instead of first-principles-based calculations will significantly reduce the resources and time required.

On the other hand, although the 500+ predicted H adsorption energies were not directly employed in the material scanning process, they provide valuable information on the adsorptive ability of bimetallic alloys and can potentially facilitate other studies on H-containing species. As mentioned in the beginning of this section, most data sets that are currently available mainly focus on C- and O-containing species, and there is a lack of relevant information on the adsorptive ability of binary alloys toward H-containing species. The DFT-calculated and ML-predicted values obtained from this work can therefore be beneficial for future research in this field.

## 4 Conclusions

In this work, a combined “Machine Learning + Density Functional Theory + Microkinetic Modelling” method was employed for the rapid scanning of bimetallic sulfur-resistant catalysts. Different ML algorithms were used to predict the atomic adsorption energy of C, H, O, and S using basic physical and chemical properties of the metallic elements and the adsorbates. Among all the ML models studied, the Ensemble learning, ANN, and SVR models achieved the best results (MAE < 1 and *R*^2^ ≥ 0.7). Results from the feature importance study demonstrated that elemental properties of the adsorbates contribute most significantly to the prediction, with the enthalpy of fusion, density, first ionization potential, and covalent radius of the adsorbate being the most important features. Over 500 bimetallic alloys were considered as candidates, obtained by the permutation between 24 elements (including transition/post-transition metals and metalloids). The adsorption energies on these bimetallic surfaces were then predicted by using the best-performing Ensemble learning model.

On the other hand, a microkinetic model was developed based on a simplified reaction mechanism of the SMR reaction, using both DFT-calculated energies and energetics obtained using the UBI-QEP method and BEP relationships. It is observed that having a carbon adsorption energy between −8.5 and −7.0 eV and an oxygen adsorption energy between −6.5 and −5.0 eV results in the highest catalytic activity for the SMR reaction. Using this range of optimal adsorption energies, the 500+ candidates were scanned, and 49 were identified to be highly active catalysts. After excluding carcinogenic and radioactive elements, the following materials were concluded to be active SMR catalysts with the best sulfur-resistant ability: Ge_3_Cu_1_, Ge_3_Ni_1_, Ge_3_Co_1_, Ge_3_Fe_1_, Rh_3_Co_1_, Rh_3_Fe_1_, Ru_3_Cu_1_, and Ni_3_Cu_1_.

In conclusion, this paper presents the first attempt to systematically scan bimetallic catalysts with high sulfur-resistant abilities. Although further improvements are possible, the ML models provided satisfying results, and the MKM greatly facilitated the scanning process. The combined “ML + DFT + MKM” method described in this work is also applicable to other chemical or pharmaceutical processes, where the rapid scanning of large material databases is required. The prediction of the sulfur-resistant materials also paves the way for future experimental studies of novel bimetallic catalysts under sulfur-containing SMR conditions.

## Data Availability

All data underlying the results are available as part of the article and in the Supporting Information file.

## Supporting Information Available

Input data set for the ML models and the MKM, predicted energies from the ML models (PDF)

## Abbreviations

ANN: artificial neural network
CV: cross-validation
DFT: density functional theory
ETR: extra trees regression
FCC: face-centered cubic
GBR: gradient boosting regression
HCP: hexagonal close-packed
KNN: K-nearest neighbors regression
Light GBM: light gradient boosting machine
MAE: mean absolute error
MKM: microkinetic model
ML: machine learning
Mol %: molar percentage
MSE: mean squared error
PAW: projector augmented wave
RFE: recursive feature elimination
RFR: random forest regression
RR: ridge regression
SMR: steam methane reforming
SVR: support vector regression
TOF: turnover frequency
