# Supplementary material for: High-Throughput Screening of Sulfur-Resistant Catalysts for Steam Methane Reforming Using Machine Learning and Microkinetic Modeling
Source: ACS Omega. 2024 Feb 28;9(10):12184–94. doi: 10.1021/acsomega.4c00119 (PMC10938427; doi:10.1021/acsomega.4c00119)
Supplement: Supplementary file 1 — ao4c00119_si_001.pdf [file ao4c00119_si_001.pdf]

Supporting Information

High throughput screening of sulphur-resistant catalysts for steam methane reforming using machine learning and microkinetic modelling

Siqi Wang[1]*, Satya Saravan Kumar Kasarapu[1], Peter T. Clough[1]*

[1] Energy and Sustainability Theme, Cranfield University, Cranfield, Bedfordshire, MK43 0AL, UK

*Corresponding author. E-mail address: P.T.Clough@cranfield.ac.uk and Siqi.Wang2019@cranfield.ac.uk

S1. Input dataset for Machine Learning model development

The 12 features used to describe the metallic elements are group, period, atomic number, atomic mass, covalent radius, Pauling electronegativity, enthalpy of fusion, density, first ionization potential, surface free energy, work function, and Wigner Seitz radius. The 9 features used to describe the adsorbates are group, period, atomic number, atomic mass, covalent radius, Pauling electronegativity, enthalpy of fusion, density, and first ionization potential.

The numerical values of the features and adsorption energies used as the input dataset for the ML model training process can be found online from the Cranfield Online Research Data (CORD) repository at https://doi.org/10.17862/cranfield.rd.24512788.v1.

S2. Input dataset for Microkinetic Model development

The elementary steps which were considered in the Microkinetic Model are listed below:

R1: $$CH_4(g) + 2* \leftrightarrow CH_3^* + H^*$$

R2: $$CH_3^* + * \leftrightarrow CH_2^* + H^*$$

R3: $$CH_2^* + * \leftrightarrow CH^* + H^*$$

R4: $$CH^* + * \leftrightarrow C^* + H^*$$

R5: $$H_2O\ (g) + 2* \leftrightarrow OH^* + H^*$$

R6: $$OH^* + * \leftrightarrow O^* + H^*$$

R7: $$C^* + O^* \leftrightarrow CO(g) + *$$

R8: $$2H^* \leftrightarrow H_2(g)$$

R9: $$C^* + OH^* \leftrightarrow COH^* + *$$

R10: $COH^* + * \leftrightarrow CO^* + H^*$

R11: $CH^* + O^* \leftrightarrow HCO^* + *$

R12: $HCO^* + * \leftrightarrow CO^* + H^*$

The atomic adsorption energies of the C, H, and O adsorbates on the seven transition metal surfaces, as well as the energies of all gas phase species were calculated using DFT. The adsorption energies of the $C_xH_yO_z$ species were obtained using the improved UBI-QEP method described in the work by Wang et al. [1]. A summary of the adsorption energies involved in the reaction model is presented in Table S1.

Table S1 Summary of the adsorption energies involved in the reaction.

	Total energy of gas phase species (eV)	Adsorption energy (eV)						
		Ni[111]	Cu[111]	Fe[110]	Pd[111]	Au[111]	Pt[111]	Rh[111]
C	-241.8	-7.86	-6.35	-8.52	-7.92	-5.56	-8.21	-8.36
H	-12.5	-3.10	-3.06	-4.21	-3.00	-2.61	-3.18	-2.17
O	-558.3	-5.36	-4.70	-6.47	-4.43	-3.09	-4.43	-4.93
CH_4	-315.5	-0.65	-0.08	-0.22	-0.17	-0.65	-0.19	-0.20
CH_3	-296.6	-1.93	-1.50	-2.13	-1.95	-1.29	-2.03	-2.08
CH_2	-277.9	-3.76	-2.70	-4.24	-3.80	-2.19	-4.01	-4.12
CH	-259.8	-5.23	-3.92	-5.82	-5.29	-3.25	-5.54	-5.68
H_2O	-597.4	-0.37	-0.75	-1.26	-0.68	-0.36	-0.68	-0.81
OH	-577.6	-2.86	-2.35	-3.75	-2.15	-1.23	-2.15	-2.53
CO	-814.4	-1.16	-1.16	-2.02	-1.76	-0.90	-1.88	-1.95
HCO	-828.8	-3.04	-2.15	-3.46	-3.08	-1.72	-3.26	-3.36
COH	-826.9	-4.45	-3.26	-4.99	-4.50	-2.67	-4.73	-4.86

The energies in the CatMAP software are computed as "relative free energies of formation" relative to a set of common reference [2]. In this study, C in CH_4, H in H_2, and O in H_2O were selected as the reference set. The equations for calculating the reference energies are listed below:

Carbon (C): $R_C = U_{CH_4} - 4U_H$

Hydrogen (H): $R_H = 0.5U_H$

Oxygen (O): $R_O = U_{H_2O} - 2U_H$

Gas phase species: $E_{C_xH_yO_z} = U_{C_xH_yO_z} - xR_C - yR_H - zR_O$

Adsorbed species: $E_{C_xH_yO_z*} = U_{C_xH_yO_z*} - U_{slab} - xR_C - yR_H - zR_O$

Where R_i is the reference energy, U_i is the DFT-calculated total energy, and E the generalized formation energy which is used in the input file for the microkinetic model. Table S2 summarises the generalized formation energies of all species involved in the microkinetic model.

Table S2 Summary of the generalized formation energies used in the microkinetic model.

	Ni[111]	Cu[111]	Fe[110]	Pd[111]	Au[111]	Pt[111]	Rh[111]
C	2.55	4.06	1.89	2.49	4.85	2.20	2.05
H	0.15	0.19	-0.96	0.25	0.64	0.07	1.08
O	2.19	2.85	1.08	3.12	4.46	3.12	2.62
CH_4	-0.65	-0.08	-0.22	-0.17	-0.65	-0.19	-0.20
CH_3	1.15	1.58	0.95	1.13	1.79	1.05	1.00
CH_2	2.21	3.27	1.73	2.17	3.78	1.96	1.84
CH	3.03	4.35	2.44	2.98	5.01	2.72	2.58
H_2O	-0.37	-0.75	-1.26	-0.68	-0.36	-0.68	-0.81
OH	1.10	1.60	0.21	1.81	2.73	1.81	1.43
CO	2.55	2.55	1.69	1.95	2.81	1.82	1.76
HCO	2.00	2.90	1.58	1.96	3.33	1.78	1.69
COH	2.51	3.70	1.97	2.46	4.29	2.23	2.10

The activation energies of the reactions were estimated using the BEP approach. The BEP relationship states that the activation barrier of a given reaction (E_a) is proportionally linked to the enthalpy of the reaction (ΔH), as shown in the equation below:

$$E_a = \alpha \cdot \Delta H + \beta$$

The classification of each reaction and the corresponding parameters used for the BEP relationship are summarised in Table S3. The parameters were extracted from the work by Wang et al. [1].

Table S3 Classification and parameters of the BEP relationship for the reactions involved in the model.

Reaction	Classification	BEP parameters (α, β)
R1	C-H bond cleavage in C_xH_y fragment	0.89, 0.75
R2	C-H bond cleavage in C_xH_y fragment	0.89, 0.75
R3	C-H bond cleavage in C_xH_y fragment	0.89, 0.75
R4	C-H bond cleavage in C_xH_y fragment	0.89, 0.75
R5	O-H bond cleavage	0.11, 0.98
R6	O-H bond cleavage	0.11, 0.98
R7	C-O bond combination	0.69, 1.99
R8	H-H bond combination	0.02, 0.91
R9	C-O bond combination	0.69, 1.99
R10	O-H bond cleavage	0.11, 0.98
R11	C-O bond combination	0.69, 1.99
R12	C-H bond cleavage in H_yCO fragment	0.89, 0.75

The microkinetic model is developed using the CatMAP software, which generates the reaction rate of a given system by solving a mean-field model to the steady state. The generalized formation energies in Table S2 and the BEP estimated activation energies using the parameters in Table S3 are used as the input data for the microkinetic model.

S3. Predicted adsorption energies

The C, H, O, and S adsorption energies on the 500+ bimetallic surfaces predicted by the ML model can be accessed online from the CORD repository at https://doi.org/10.17862/cranfield.rd.24486466.

S4. Promising catalysts identified by the screening process

The promising candidates for the SMR reaction identified by the Microkinetic Model are listed in Table S4.

Table S4 Catalysts with optimal performance identified through the screening process.

	**Bimetallic catalyst with a M1:M2 molar ratio of 3:1**		**Adsorption energy (eV)**		
**No.**	**M1**	**M2**	**C**	**O**	**S**
1	Ge	Cu	-7.62650	-5.01768	-5.02157
2	Ge	Ni	-7.67237	-5.08801	-5.12017
3	Rh	Co	-7.30665	-5.14343	-5.18526
4	Rh	Fe	-7.17542	-5.16505	-5.30687
5	Ge	Co	-7.50246	-5.17161	-5.33429
6	Ni	Cu	-7.67273	-5.10829	-5.43571
7	Ge	Pd	-8.16988	-5.00004	-5.44785
8	Ge	Fe	-7.34338	-5.15616	-5.45589
9	Tc	Ni	-8.17942	-5.05062	-5.50654
10	Ru	Cu	-8.03899	-5.10794	-5.59828
11	Co	Cu	-7.67982	-5.19725	-5.68112
12	Ru	Ni	-8.11078	-5.22111	-5.69688
13	Tc	Co	-8.16705	-5.21196	-5.72066
14	Ni	Co	-7.57855	-5.3748	-5.74842
15	Co	Ni	-7.70955	-5.27347	-5.77972
16	Ge	Rh	-7.94664	-5.1144	-5.79988
17	Tc	Fe	-8.09804	-5.23099	-5.84227
18	Zn	Rh	-7.04996	-5.0047	-5.8551
19	Ni	Pd	-8.40054	-5.11331	-5.86198
20	Mo	Co	-8.48787	-5.11887	-5.86362
21	Ni	Fe	-7.44876	-5.40117	-5.87003
22	Fe	Cu	-7.98197	-5.24044	-5.90131
23	Ru	Co	-8.06237	-5.39127	-5.911
24	Cu	Rh	-7.42594	-5.12412	-5.91599
25	Mo	Fe	-8.42501	-5.12581	-5.98522
26	Pd	Ru	-7.21282	-5.26812	-5.9991
27	Fe	Ni	-7.99754	-5.3142	-5.99991
28	Ru	Fe	-7.95624	-5.40468	-6.0326
29	Co	Pd	-8.45703	-5.19666	-6.1074
30	Co	Fe	-7.53317	-5.51052	-6.11544
31	Rh	Tc	-7.26201	-5.06673	-6.20417
32	Ni	Rh	-8.11137	-5.29894	-6.21402
33	Fe	Co	-7.95683	-5.52296	-6.21403
34	Rh	Ru	-7.67187	-5.50068	-6.23871
35	Ge	Tc	-7.22822	-5.03607	-6.35319

36	Ge	Ru	-7.48506	-5.29524	-6.38774
37	Co	Rh	-8.18295	-5.39004	-6.45943
38	Cu	Ru	-7.01998	-5.48503	-6.50385
39	Fe	Rh	-8.45292	-5.4239	-6.67963
40	Ni	Tc	-7.30862	-5.2763	-6.76733
41	Tc	Ru	-8.47608	-5.56946	-6.77411
42	Ni	Ru	-7.58614	-5.64244	-6.80187
43	Ru	Tc	-7.96193	-5.4105	-6.9299
44	Co	Tc	-7.45794	-5.37037	-7.01274
45	Co	Ru	-7.69492	-5.74318	-7.04729
46	Ru	Mo	-7.47125	-5.13888	-7.21184
47	Fe	Tc	-7.80207	-5.40444	-7.23293
48	Fe	Ru	-7.96675	-5.76451	-7.26748
49	Fe	Mo	-7.17289	-5.06135	-7.51487

S5. Adsorption sites considered for the mono and bimetallic surfaces

The eight high symmetry sites considered for the mono and bimetallic surfaces are illustrated in Figure S1.

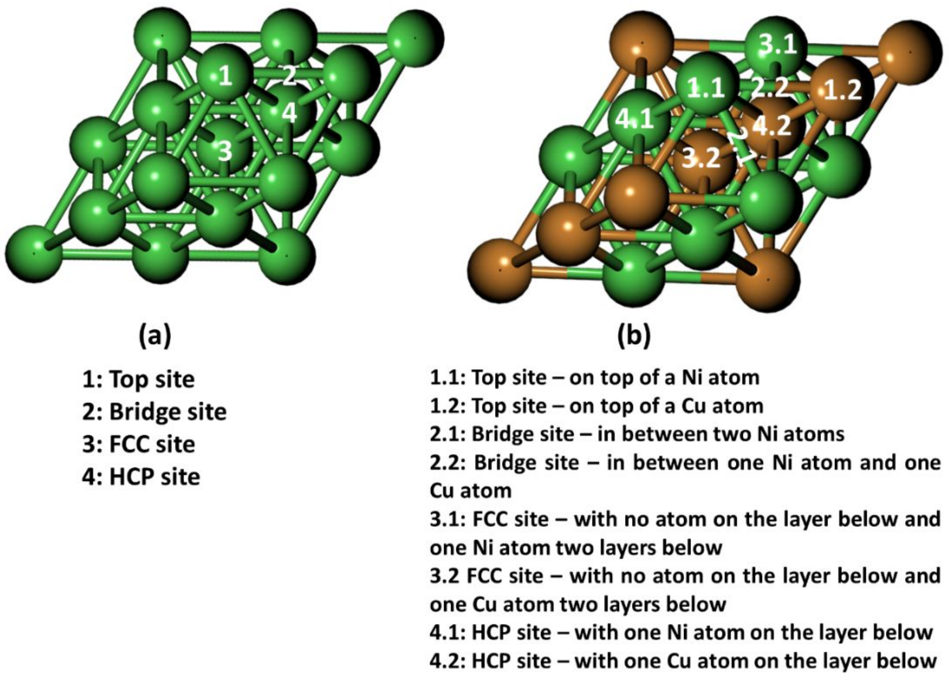

Figure S1 Location of adsorption sites.

References

[1] Wang Y, Xiao L, Qi Y, Mahmoodinia M, Feng X, Yang J, et al. Towards rational catalyst design: Boosting the rapid prediction of transition-metal activity by improved scaling relations. Physical Chemistry Chemical Physics 2019;21:19269–80. https://doi.org/10.1039/c9cp04286e.

[2] Medford AJ, Shi C, Hoffmann MJ, Lausche AC, Fitzgibbon SR, Bligaard T, et al. CatMAP: A Software Package for Descriptor-Based Microkinetic Mapping of Catalytic Trends. Catal Letters 2015;145:794–807. https://doi.org/10.1007/s10562-015-1495-6.
